# A United Kingdom nationally representative survey of public attitudes towards pharmacogenomics

**DOI:** 10.1093/qjmed/hcaf035

**Published:** 2025-02-20

**Authors:** Emma F Magavern, Gabriel Marengo, Participant Panel at Genomics England, Emma F Magavern, Gabriel Marengo, Chujan Sivathasan, Marta Mezzanzanica, Alison J Wright, Jessica Keen, Videha Sharma, John H McDermott, Claire Duckett, Dave McCormick, Shelley Simmonds, Emma Walters, John Weinman, Vivienne Parry, William G Newman, Mark J Caulfield, Chujan Sivathasan, Marta Mezzanzanica, Alison J Wright, Jessica Keen, Videha Sharma, John H McDermott, Claire Duckett, Dave McCormick, Shelley Simmonds, Emma Walters, The National Centre for Social Research Team, Emma F Magavern, Gabriel Marengo, Chujan Sivathasan, Marta Mezzanzanica, Alison J Wright, Jessica Keen, Videha Sharma, John H McDermott, Claire Duckett, Dave McCormick, Shelley Simmonds, Emma Walters, John Weinman, Vivienne Parry, William G Newman, Mark J Caulfield, John Weinman, Vivienne Parry, William G Newman, Mark J Caulfield

**Affiliations:** William Harvey Research Institute, Queen Mary University of London, London, UK; William Harvey Research Institute, Queen Mary University of London, London, UK; Genomics England Ltd, London, UK; The National Centre for Social Research, London, UK; The National Centre for Social Research, London, UK; School of Cancer and Pharmaceutical Sciences, Faculty of Life Sciences and Medicine, King’s College London, London, UK; NHS North West Genomic Medicine Service Alliance, UK; Division of Informatics, Imaging and Data Science, School of Health Sciences, Faculty of Biology, Medicine and Health, University of Manchester, Manchester, UK; Manchester Centre for Genomic Medicine, St Mary’s Hospital, Manchester University Hospitals NHS Foundation Trust, Manchester, UK; Division of Evolution, Infection and Genomics, School of Biological Sciences, The University of ′Manchester, Manchester, UK; Genomics England Ltd, London, UK; Genomics England Ltd, London, UK; Genomics England Ltd, London, UK; Genomics England Ltd, London, UK; Genomics England Ltd, London, UK; School of Cancer and Pharmaceutical Sciences, Faculty of Life Sciences and Medicine, King’s College London, London, UK; Genomics England Ltd, London, UK; Manchester Centre for Genomic Medicine, St Mary’s Hospital, Manchester University Hospitals NHS Foundation Trust, Manchester, UK; Division of Evolution, Infection and Genomics, School of Biological Sciences, The University of ′Manchester, Manchester, UK; William Harvey Research Institute, Queen Mary University of London, London, UK

## Abstract

**Background:**

Variation in DNA is known to contribute to medication response, impacting both medicine effectiveness and incidence of adverse drug reactions (ADRs). However, clinical implementation of pharmacogenomics (PGx) has been slow, and the views of the public are not well understood.

**Aim:**

To assess UK national public attitudes around pharmacogenetics.

**Design and Methods:**

The survey was co-designed with the Participant Panel at Genomics England and the data were collected by the National Centre for Social Research, using its nationally representative panel of UK adults. Multivariable logistic regression analyses were used to analyse relationships between selected survey reported variables, controlled for age and sex.

**Results:**

The survey response rate was 58%. Two thousand seven hundred and nineteen responses were obtained. Most respondents (59%) had experienced either no benefit or a side effect. Forty-five per cent of respondents reported having experienced no benefit and 46% of respondents reported having experienced a side effect, with female respondents more likely to be in both groups (*P* < 0.0001). Despite variability in interindividual medicine response being well understood (89%), the involvement of DNA in predicting benefit or risk of a side effect is not (understood by 52% and 48%, respectively). Eighty-nine per cent would complete a PGx test, with 91% wanting direct access to this information. Eighty-five per cent of UK adults think that the NHS should offer PGx to those regularly taking many medicines. Respondents were not more worried overall about misuse of PGx data compared with other routine medical data. Experience with prescription medication impacted on views with those who were prescribed medication almost twice as likely to want a PGx test for any reason.

**Conclusion:**

Most respondents reported experience with either a medication not working for them or ADRs. There was a high level of understanding of variable medication response but a relatively low level of awareness of the role genetics plays in that variability. Most respondents would want a PGx test, to have direct access to results, and think the NHS should offer this form of testing. Importantly, respondents were not more concerned about PGx data use than that of any other routinely generated medical data. Notably, this study highlights a relationship between individuals’ experiences with prescription medications and their interest in PGx testing, underscoring the potential for personalized medicine to address public healthcare needs.

## Graphical Abstract

**Figure:**
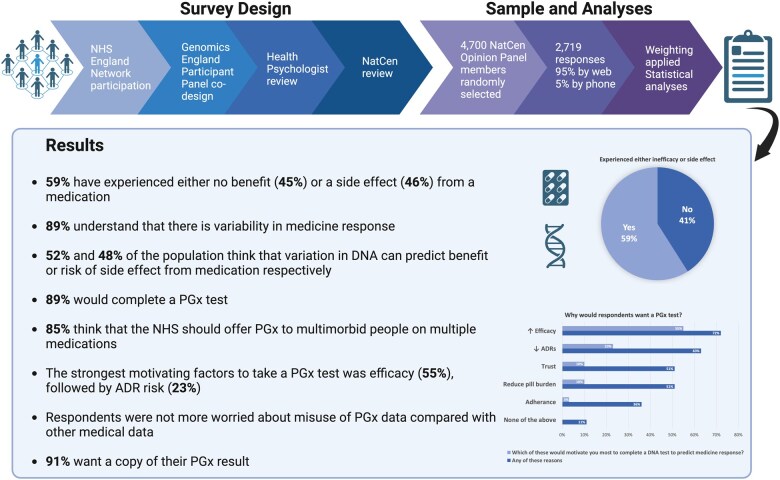

## Introduction

Population-level data show that an increasing number of medications are being prescribed in the United Kingdom (UK) and that people are more likely than ever to take many medications.^1^^,^^2^ There is variability in response to medications, with some people suffering side effects or not deriving the intended benefit. Variation in DNA is known to contribute to this interindividual variability in medication response.^3^ This field of study is referred to as pharmacogenomics (PGx).

PGx testing holds significant potential for improving medication safety and efficacy by tailoring drug choices and dosages to individual genetic profiles. Current guidelines, such as those developed by the Clinical Pharmacogenetics Implementation Consortium, already support clinical application for many drug–gene interactions.^4^ Large-scale biobanks, such as the UK Biobank, have demonstrated the prevalence of clinically actionable pharmacogenetic variants across diverse populations, with nearly all individuals carrying variants that could affect drug response.^5^^,^^6^

Though decades of research have revealed many well-validated gene–drug pairs, with prospective clinical trial evidence of benefit from personalizing therapy based on variants in DNA to reduce risk of an adverse reaction or ineffectiveness, clinical implementation has lagged behind the science.^7^ Substantial factors contributing to this slow uptake in clinical practice include operational challenges in fitting a nondiagnostic form of genetic testing into clinical pathways at scale for commonly prescribed medications, incorporation of PGx into standard clinical informatics pathways and electronic health records, transfer of information between care settings, upskilling the prescribing workforce, and, importantly, low patient and public awareness of PGx.^3^^,^^8^^,^^9^

As the UK increasingly integrates genetic data into healthcare, PGx testing is anticipated to play a critical role in reducing adverse drug reactions and optimizing treatments, particularly as genetic testing becomes more accessible and affordable.^5^^,^^6^ There has been increasing attention placed on the need to accelerate adaptation of pharmacogenomics, with a joint report from the British Pharmacological Society and the Royal College of Physicians making a range of recommendations to overcome recognized barriers, and a shared emphasis in government and NHS policy publications.^10–12^

To our knowledge, there has been no national scale public survey or consultation of attitudes around pharmacogenomics in the UK.

There is considerable urgency to gather such feedback from the public in the UK due to the emergence of new implementation drivers. These drivers include national guidance for *CYP2C19* genetic testing to optimize medical therapy after ischemic stroke and regulatory requirements to test the same gene to guide dosage with the first in class myosin inhibitor, mavacamten (indicated to treat hypertrophic obstructive cardiomyopathy).^13–15^ At the same time, regulators are increasingly adding PGx information to drug labels.^15^

The *CYP2C19* gene encodes cytochrome P450 2C19 (CYP2C19), a key hepatic enzyme common to the metabolism pathway of many different medications prescribed across general medical practice and clinical specialism silos. These include clopidogrel, many antidepressants and antipsychotics, proton pump inhibitors, and other medications.^16^^,^^17^  *CYP2C19* is one of three genes (along with *CYP2D6* and *SLCO1B1*) which both cover the largest volume of potential prescribing interventions based on longitudinal English prescribing data and interact with the most commonly reported causes of PGx mitigatable adverse drug reactions nationally based on Yellow Card (YC) data from a 50-year period.^18^^,^^19^

There is a lack of clear lay information available around PGx and well-documented concerns that people often do not understand PGx results when they receive them.^20^^,^^21^ Consensus around consent for testing is lacking, and current practices are heterogenous.^22^^,^^23^

There is a limited understanding of patient priorities for a theoretical new PGx service. In the context of a publicly funded UK National Health System, it is critical to canvas public opinion. There is also a dearth of policy and legislation specific to PGx, which has different implications from diagnostic genetic testing.^8^ Though use of genetic markers in PGx is to optimize medication use, similar to the use of liver or renal function tests, population-scale PGx testing would generate additional information about population and family structure. The implications and possible consequences of clinical pathways generating and potentially sharing these data for future research are not yet understood. Questions around responsibility, uncertainty, risk acceptability, consent, and data sharing need to be posed to the public as they are normative questions about societal acceptability.

To address this gap in the evidence, we have therefore undertaken a probability-based panel survey to represent views of the public in the UK towards pharmacogenomics, in partnership with the National Centre for Social Research (NatCen).

### Objective

To assess national public attitudes around the routine use of DNA testing to predict medicine response.

## Materials and methods

### Survey design

A survey was designed in collaboration with pharmacogenomics expert stakeholders as part of the NHS England Network of Excellence for Pharmacogenomics and Medicines Optimisation to ensure face validity. The Participant Panel at Genomics England co-designed and tested the survey questions, and they were reviewed by academic experts in health psychology and experts in survey design within the NatCen team. The complete survey with data collection specifications forms Supplement S1. The survey was scripted using NatCen’s established survey template, with UNICOM Intelligence software.

### Data collection

NatCen was commissioned to collect data using the NatCen Opinion Panel, its nationally representative random probability-based panel of adults at least 18 years old in the UK.^24^ Data collection took place from July 5 to August 4 2024 with web and telephone interviews. To ensure the achieved sample of respondents was representative of the population, a set of non-response weights was computed to account for non-response to the recruitment surveys, refusal to the join the panel, and non-response in the survey of panel members itself. The use of complex survey design metrics (namely: weights, strata, and primary sampling unit) in the analysis ensured the representativeness of the results to the UK population. Demographic data had been updated within 6 months of the survey, with the exception of relatively ‘static’ variables such as ethnicity or sex.

### Recruitment and sampling

Participants were recruited from NatCen’s probability-based panel of UK adults (aged 18 and above). For this survey, all panel members recruited from the British Social Attitudes (BSA) survey (from 2015 onwards) and the Life in Northern Ireland (NLI) survey, who had not subsequently left the panel or become inactive (i.e. did not take part in any of the last 6 surveys they were invited to), were eligible to be invited. Of these, a random sub-sample (*n* = 4700 cases) was selected, maintaining the probability-based design.

Using a model that incorporates panel members’ age, sex, region, household structure, income, education, economic activity, ethnicity, housing tenure, social class, interest in politics and party support, selection odds for inclusion in the survey were modified to improve the representativeness of the sample. Selection odds were also adjusted through overrepresentation in Wales and Northern Ireland to enable analysis of responses within those countries.

### Fieldwork design

Fieldwork followed a sequential mixed mode design. Panel members were initially invited to participate in the research online, and sent multiple reminders by post, emails and/or text message. If they had not completed the interview after two weeks (and if telephone numbers were available), they were then contacted by NatCen’s Telephone Unit to encourage online completion or offer an interview over the phone (landline or mobile). In this way, we were able to include those who are unable or unwilling to complete online. A £5 Love2Shop voucher was sent as a ‘thank you’ to those who participated. The fieldwork period lasted for 31 days. Although most participants completed within the first week, this ensured that everybody had the opportunity to take part, and not only those that are ‘readily’ available. Web fieldwork ran from 5 July to 4 August and telephone fieldwork ran from 14 July to 4 August. To improve sample quality, fieldwork resources were balanced away from those who are typically over-represented in the sample and that take part regularly and targeted at those who are underrepresented in the sample and take part less regularly.

### Weighting

The weights used to adjust for nonresponse are described in Supplement S2.

### Statistical analysis

All analyses were done in R studio.^25^ The survey package was used.^26^ The weighted results are presented in this manuscript, with the strata and primary sampling unit included in the survey design function for statistical analyses presented. The adjusted Wald test was used to compare discrete variables for subgroup analyses. Due to reports of sex differences in background literature, we analysed experience with medication ineffectiveness or ADRs by sex. Multivariable logistic regression analyses were used to analyse relationships between selected survey reported variables, controlled for age and sex. Our chosen variables were reflective of the literature, probing association between medication experience and PGx testing desirability and motives, as well as ADR experience, awareness of ADR reporting system, and desire for PGx information integration into ADR reports.

## Results

Survey respondent demographics are shown in Table 1. The survey response rate was 58%. Two thousand seven hundred and nineteen responses were obtained, 95% through web interviews, with the remaining 5% from telephone interviews. Full survey results are available in Supplement S3.

**Table 1:** Survey respondent demographic information shown with percentage on the *x* axis

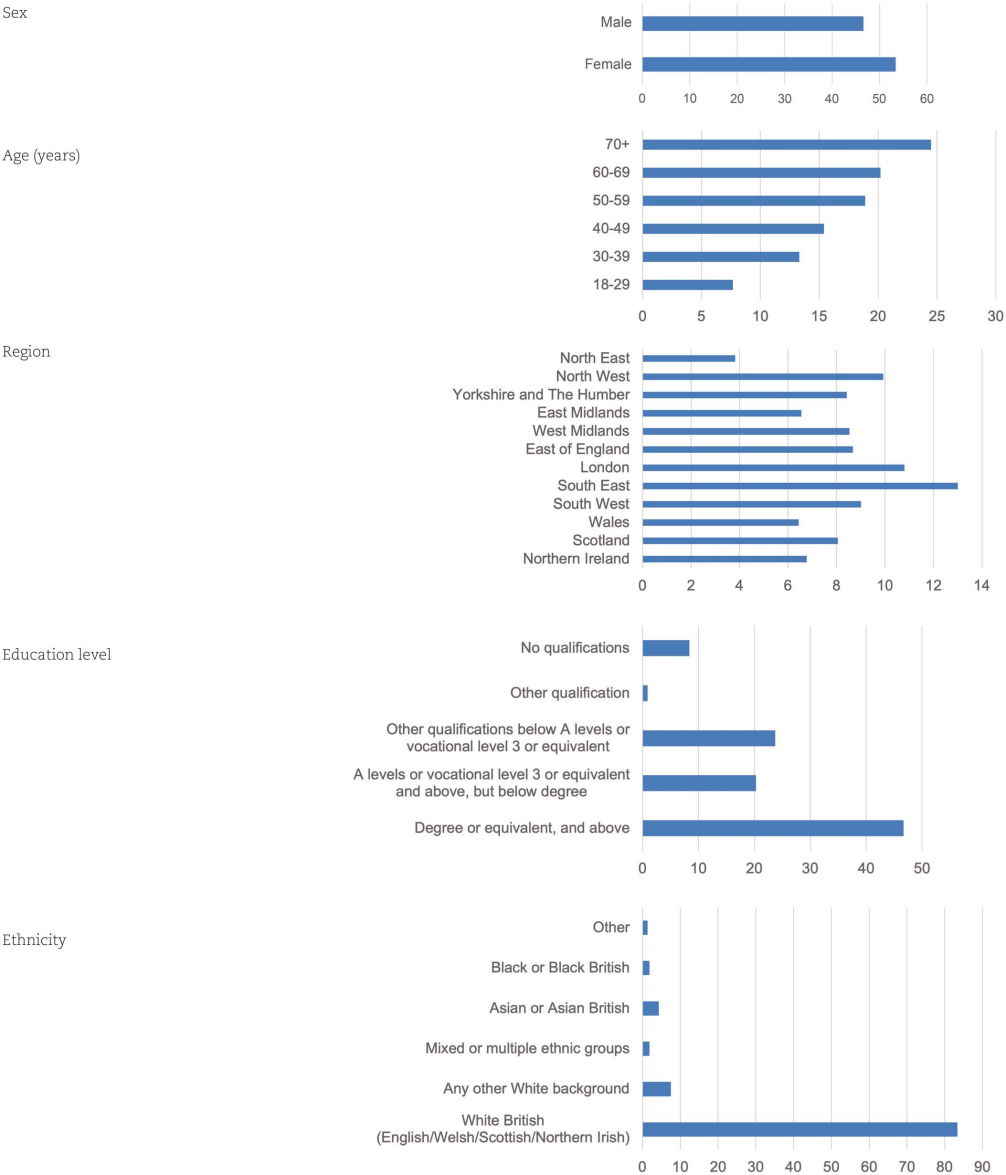
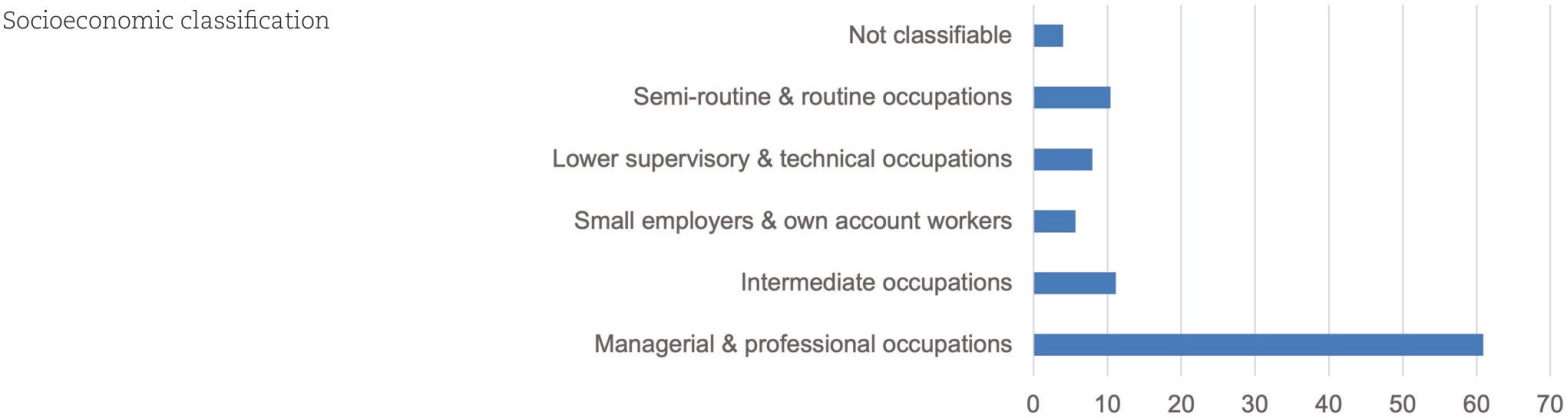

### Experience with medication and understanding of PGx

Over half of participants (56%) were prescribed medicines regularly, with an average of four medications prescribed. Most respondents (59%) had experienced either no benefit or a side effect from a medication. Forty-five per cent of respondents had experienced no benefit, and 46% of respondents had experienced a side effect from a medication. More female respondents reported both medicines not working for them (*P* < 0.0001) and having experienced side effects from medications (*P* < 0.0001).

Most respondents (89%) disagreed that everyone responds to medicine in the same way, but only about half of participants thought that variation in DNA can predict either benefit (52%) or risk of a side effect (48%) from a medication, while 45% and 49% of participants, respectively, said they did not know if this was the case. Male sex, younger age group, and higher level of education were significantly associated with awareness of the role of pharmacogenomics to predict both ADR (male sex OR 1.76, CI 1.46–2.14, *P* < 0.0001, increasing age category OR 0.45, CI 0.35–0.59, *P* < 0.0001, lower level of education category OR 0.57, CI 0.39–0.83, *P* = 0.0036) and benefit from a medication (male sex OR 1.43, CI 1.18–1.73, *P* = 0.0002, increasing age category OR 0.69, CI 0.54–0.90, *P* = 0.006, lower level of education category OR 0.50, CI 0.35–0.71, *P* = 0.0001).

### Desirability of PGx testing

**Figure 1.**
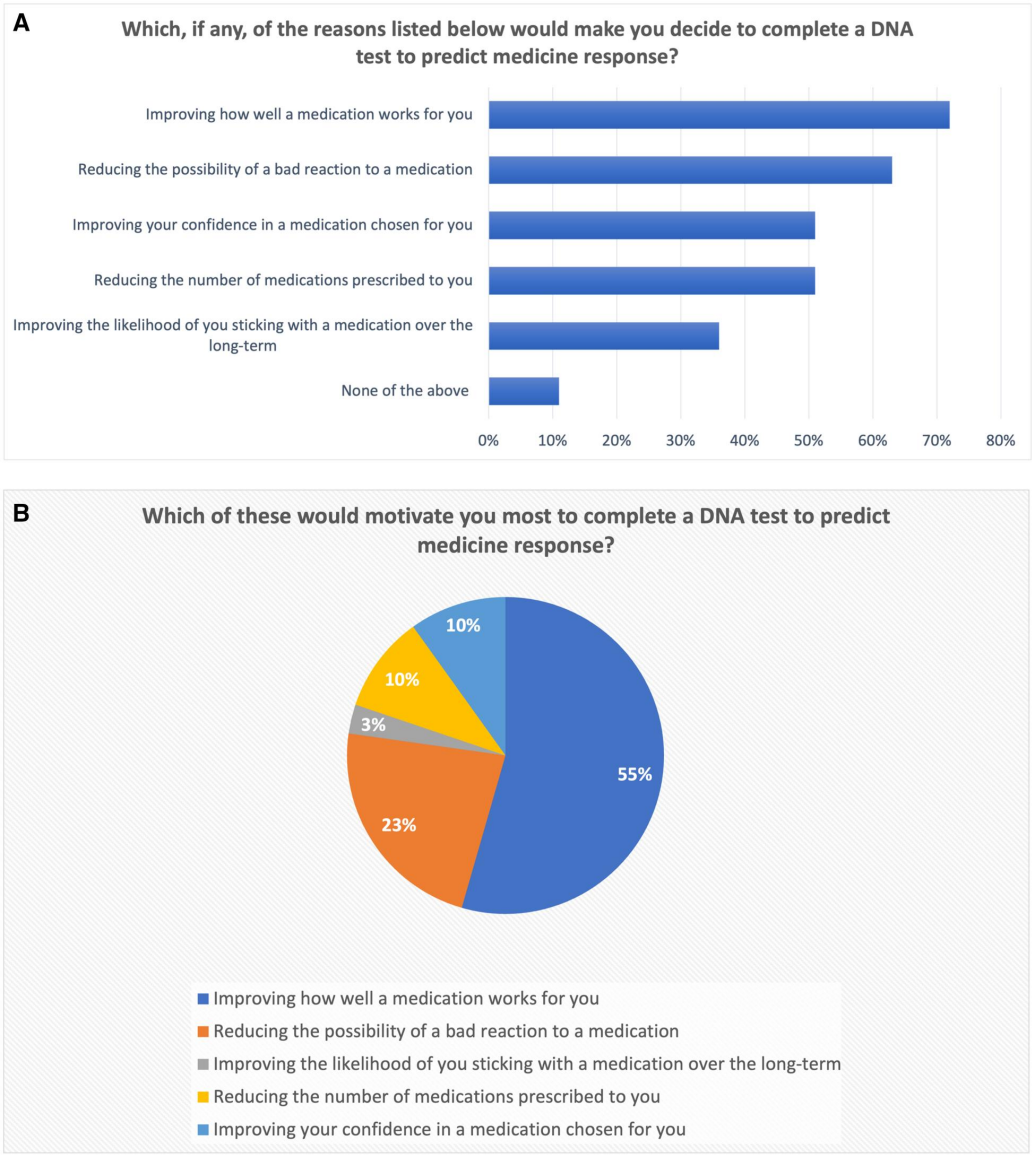
(A) Motivations to undertake PGx testing. (B) Strongest motivation to undertake PGx testing.

Notably, 89% of participants said they would complete a PGx test (Figure 1A). Experience with prescription medication impacted on attitudes with those who were prescribed medication almost twice as likely to want a PGx test for any reason (OR 1.94, CI 1.42–2.65, *P* < 0.0001). The most commonly endorsed motivations for PGx testing were to enhance drug effectiveness (72%) and reduction of ADR risk (63%; Figure 1A and B). Those who reported experiencing a side effect from a medication were significantly more likely to select reduction of ADR risk as a motivating factor for PGx testing (OR 1.74, CI 1.43–2.13, *P* < 0.0001). Likewise, those who reported having experienced medication ineffectiveness were more likely to be motivated to take a PGx test by a promise of increased effectiveness (OR 1.25, CI 1.01–1.55, *P* = 0.04).

**Figure 2.**
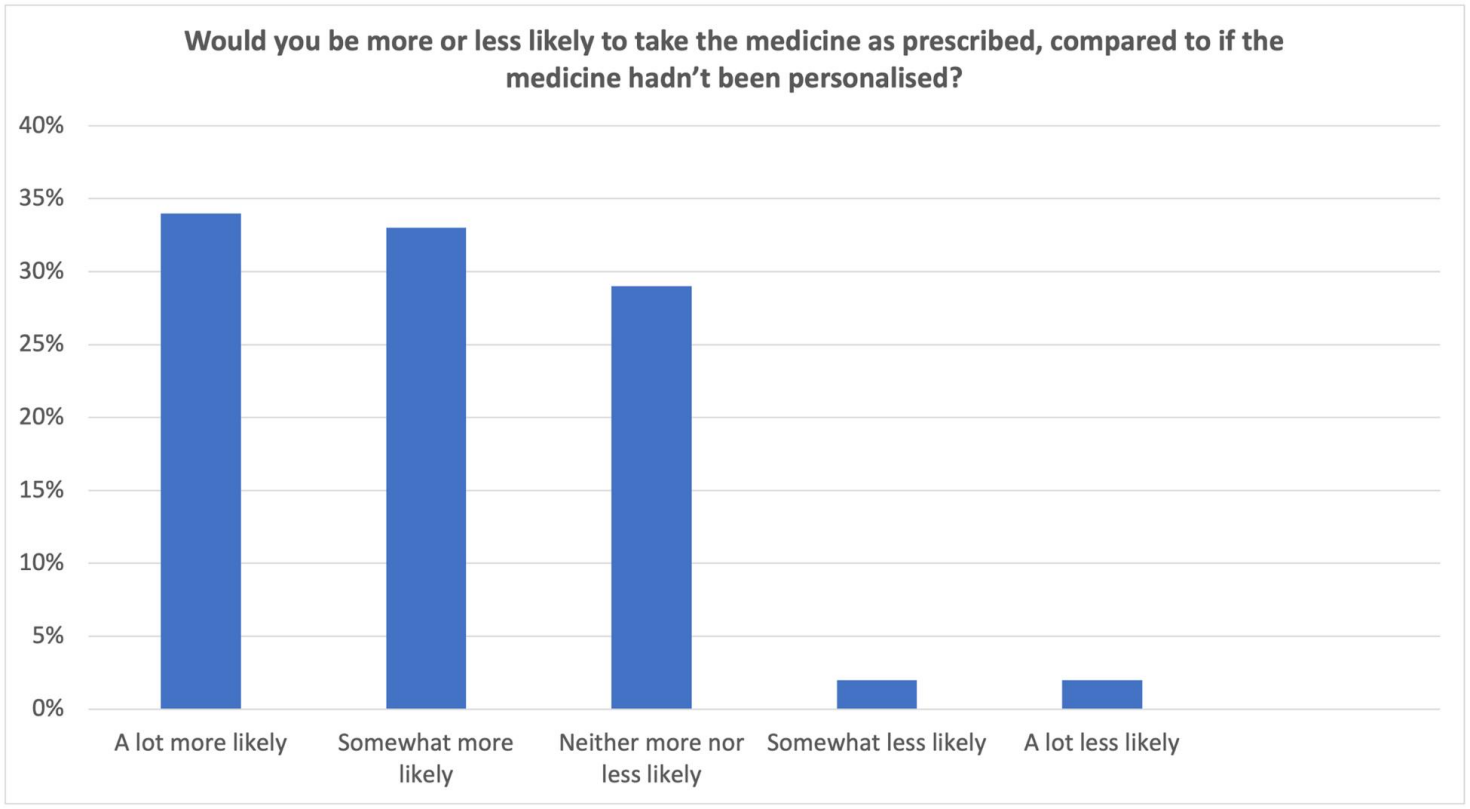
Perceived impact of PGx on medication adherence.

Sixty-seven per cent of UK adults reported that having a PGx test, and their prescription adjusted accordingly, was likely to increase their medication adherence, compared to if the medicine had not been personalized (Figure 2). Eighty-five per cent of UK adults thought that the NHS should offer PGx to people with multimorbidity prescribed many medications, while 58% of UK adults thought that the NHS should offer PGx to everyone.

### Patient information needs

Most participants responded that it was “very important” to make patients aware of certain information prior to consenting to a pharmacogenomic test, including that PGx is a form of genetic testing (52%), that it is intended to make medicines safer and more effective (73%), that even with PGx testing the patient may still have an ADR (72%) or experience lack of effectiveness (67%). Ninety-three per cent had not encountered any patient materials explaining PGx. Participants wanted this information from trusted health care professional sources (79% via leaflets and 73% posters at GP practices or hospital clinics). Free text comments also suggested a desire for information directly from medication prescribers or dispensers. About a third of UK adults would like online videos (31%) or social media platforms (32%) as a source of PGx information.

### Data stewardship

With regard to data, people overwhelmingly (91%) wanted access to their own PGx data as well as agreeing that these results should be stored in their medical records (91%). Seventy-eight per cent agreed that PGx results should be available in the NHS app.

### Concerns about pharmacogenomic data

**Figure 3.**
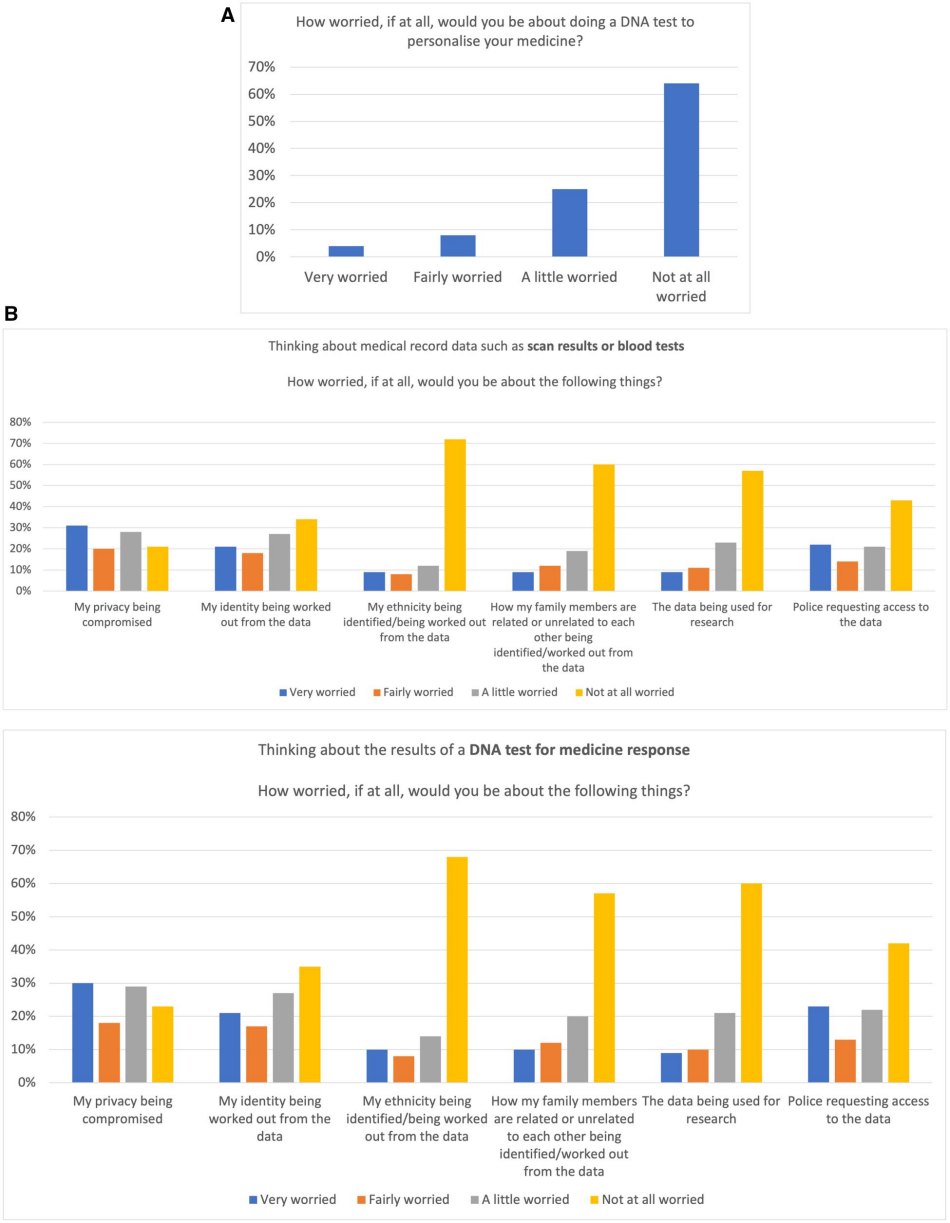
(A) Concerns about PGx testing. (B) Concerns about PGx data as compared with other routinely generated clinical data.

Sixty-four per cent were not at all worried about having a PGx test, while 25% were a little worried. Few participants were fairly (8%) or very worried (4%; Figure 3A). When potential causes for concern were compared between uses of routinely collected health care data and PGx data, results were very similar. With pharmacogenomics, there was a marginally higher concern about police requesting access to PGx data (1% more worried, *P* < 0.0001), ethnicity identification (3% more worried, *P* < 0.0001), or identification of family relatedness (2% more worried, *P* < 0.0001) as compared with routine health care data (Figure 3B). In other areas, respondents reported less worry about PGx data as compared with other routine health care data (privacy 2% less worried, *P* < 0.0001, identifiability 1% less worried *P* < 0.0001, use of data for research 3% less worried, *P* < 0.0001).

### Data sharing

Most participants were happy for PGx data to be shared for research purposes with health care professionals (82%) and medical regulators (76%), with 59% happy to share the data with academics. Twenty-five per cent were willing to share the data with charities or non-profit organizations, with only 10% of UK adults willing to share data with private companies and 9% not willing to share data for research with any of these groups.

Only 25% of participants were aware of the YC national system for reporting ADRs.^27^ Of those, 88% knew that anyone could submit a YC report. However, 69% of UK adults would like the option to include DNA test results in any ADR report. Those participants who reported having experienced an ADR were almost twice as likely to be aware of the YC ADR reporting system (OR 1.94, CI 1.58–2.39, *P* < 0.0001), and participants who were aware of the YC ADR reporting system were more likely to want to include PGx data in an ADR report made to the UK national medicines regulator, The Medicines and Healthcare products Regulatory Agency (MHRA) (OR 1.33 CI 1.05–1.68, *P* = 0.02).

## Discussion

This high response rate survey of a representative sample of the UK population shows how commonly UK adults had experienced a medication not working for them or adverse reactions. It shows a high level of understanding of variable medication response from lived experience but a relatively low level of awareness of how genetics contributes to such variability, highlighting the need for visible public dialogue around PGx. Most participants felt that the possibilities of enhancing medication effectiveness or reducing ADRs would make them decide to have a pharmacogenomic test.

The fact that respondents who were prescribed regular medicines were more likely to want PGx testing shows the relationship between lived experience with medication and a desire for a more targeted prescribing approach incorporating DNA variants. This is shown in a more granular way by the significant relationship between having experienced an ADR or ineffectiveness and choice of these factors as motivations for PGx testing.

It is a useful metric of public opinion to see that most respondents would want a PGx test and think the NHS should offer this form of testing, with especially strong endorsement for patients with multimorbidity prescribed multiple medicines. Interestingly, effectiveness was a greater priority for respondents than decreasing ADR risk, despite the sampled group having had similar frequency of experience of ADRs and ineffectiveness. Though enhancing medication adherence was not a high priority in various motives for PGx testing, more than two in every three respondents did think that PGx testing was likely to improve their medication adherence compared to non-personalised prescribing. It remains to be assessed if this is what happens in practice during PGx implementation. Prior research is inconclusive, with some studies finding a potentially positive impact on adherence and some finding no effect.^28^^,^^29^ Influences on medication are multifactorial, and PGx will only alter some of them.^30^

Survey respondents have given us an empiric basis for components of informed consent with agreement that patients needed to be aware of all proposed aspects of consent, including that PGx is a form of genetic testing, that it is intended to make medicines safer and more effective, and that, even with PGx testing, the patient may still have an ADR or experience lack of effectiveness. This can form the basis for a standardised minimum information provision for informed consent. Findings demonstrate a dearth of available PGx lay information materials, which should be addressed with funded dedicated work streams to accompany any implementation initiatives. In the NHS, healthcare provider PGx information needs are being addressed with the development of GeNotes, which is a free publicly available education resource curated by NHS England’s Genomic Education Programme in collaboration with topic experts and includes a pharmacogenetics section.^31^ However, although GeNotes signposts to existing resources for patients, it does not create lay resources. A similar national initiative dedicated to generating high-quality patient information would be fruitful and is needed.

Importantly, comparing concerns about routine medical data with PGx data shows that on balance the public is no more concerned about PGx than any other routinely generated data. Our survey showed there were slightly elevated levels of concern about a few aspects of PGx testing, namely police requesting access to data, and identification of ethnicity and family relatedness. These are important areas to address in a policy forum, as national level implementation of PGx would indeed give information about population and family structure and be of obvious forensic interest. We have already seen examples of direct-to-consumer ancestry genetic data being used by police to locate and prosecute criminals.^32^

The overwhelming majority of respondents (91%) wanted access to their own PGx results. This is consistent with prior findings showing that public acceptability of PGx is driven by patient access to their own data and highlights the need for further work to ensure that results are presented in a form that is readily understood.^33^

Concerns around health data ownership and data sharing are well documented and most recently laid bare in the UK by the Care.data and NHS digital opt out arrangements.^34–36^ It is clear from these survey responses that access gating and research permissions for population-level PGx testing would need to be agreed on a national level with corresponding policy prior to implementation. It is striking that 90% of UK adults were not willing to share data with private companies and 9% were not willing to share data for research with any organisations. This must be further explored, and these wishes respected proactively and prospectively. Prior qualitative work with British South Asian ancestry focus groups suggested specific concerns around data exploitation and misuse by private industry.^37^ Our finding that most respondents were happy for PGx data to be shared for research purposes with health care professionals (82%), the medicines regulators (76%), and academics (59%) correlates with a recently published discrete choice experiment, which shows that approximately three quarters of respondents were happy for their genetic data to be shared across the NHS for care and research.^33^

The MHRA, is piloting a biobank where genetic architecture of reported ADRs could be elucidated.^38^ There is a clear opportunity for clinical pharmacogenomics to dovetail with enhanced pharmacovigilance where clinically generated PGx data could be shared with regulators as part of any adverse event reporting on a national scale. In the context of this initiative, it was encouraging to see that most respondents would like PGx results included in national ADR reports. The fact that respondents who were aware of the reporting system were more likely to want to have PGx results included suggests that, if awareness of the MHRA pharmacovigilance systems were increased, more people may wish to include these data.^38^ Overall, low awareness of the YC reporting system shows a large margin for improvement in public awareness and it is unsurprising that participants who had experienced an ADR were more likely to be aware of the YC ADR reporting system. The heightened awareness of PGx in the younger age groups and those with higher levels of education points at opportunities for targeted PGx awareness raising campaigns.

The finding that female respondents reported higher rates of both ADRs and ineffectiveness is in keeping with other reports in published peer-reviewed literature.^39–41^ Studies have not yet revealed if there are significant differences between men and women in either perceived or proven benefits of implementing PGx.

### Study strengths and limitations

Strengths of this study include the high-quality probability-based panel design to garner data from a representative subset of UK residents, high response rate and number of respondents, and united expert and lay person stakeholder input on question content, design, and format. A limitation is that there are no validated tools to measure attitudes towards pharmacogenomics, and therefore none were used in this study.

## Conclusion

Over 2,700 respondents to a nationally representative probability-based panel survey with a 58% response rate in the UK were positive about pharmacogenomics implementation, with most respondents supporting integration in the NHS, indicating that they would want a PGx test, and not expressing more concerns as compared with other routine medical tests. However, there is a lack of available patient information at present and some specific concerns about potential data misuse that require mitigation prior to implementation. These include access to clinically generated PGx data for research, particularly by private entities, and potential identification of family and population structure by PGx data, as well as police requests for these genetic data.

### The patient perspective

This survey’s findings reinforce what we on the Participant Panel at Genomics England have always known and advocated for: that the majority of people support the move towards truly individualised health care. People want control of their own health and importantly access to their own data. We note the overwhelming number (91%) who wanted access to their PGx data. Patients want to be partners in their health care. This research opens the doors for further public engagement on implementation, particularly interpretation of the results of PGx testing. It also points to the need for greater awareness of PGx testing, starting with the development with patients of a clear and coherent set of messages. Beginning this journey in primary healthcare settings will need to be thought through and properly planned. It will be important not to over-promise on any outcomes that may come from it, learning lessons from projects such as the 100 000 Genomes Project and its additional findings programme. This survey is a great first building block for the future of PGx.

## Supplementary Material

hcaf035_Supplementary_Data

## Data Availability

All relevant data have been made available openly in the Supplementary materials provided. The full raw data set will be made available by NatCen after publication.

## PPIE involvement statement

The Participant Panel at Genomics England co-designed this survey in a workshop format led by Vivienne Parry. The panel/members then reviewed the analysis and contributed to the manuscript with a patient perspective.
