# Supplementary material for: A United Kingdom nationally representative survey of public attitudes towards pharmacogenomics
Source: QJM. 2025 Feb 20;118(6):423–33. doi: 10.1093/qjmed/hcaf035 (PMC12419794; doi:10.1093/qjmed/hcaf035)
Supplement: hcaf035_Supplementary_Data [file hcaf035_supplementary_data.zip › Supplement 2 Weighting.docx]

*Weighting*

Non-response to NatCen Opinion Panel surveys can occur at various points: the recruitment survey, the invitation to join the panel (at the end of the recruitment survey), subsequent attrition from the Panel, and the survey of panel members itself. The recruitment surveys are already weighted to adjust for non-response. Further weights were computed to adjust for non-response at the subsequent stages. The final weight used in the analysis is the product of these weights. This multi-stage approach is ideal because the correlates of non-response can be different at each stage.

The three weights computed are:

1. *Recruitment survey weight*: The weights from the recruitment surveys followed similar

designs. They comprise three components: selection weights to adjust for uneven selection probabilities; non-response weights computed via logistic regression models of response (at address level) to adjust for differential non-response; and calibration to population estimates. Panel members invited to take part in this study on pharmacogenetic were recruited from the British Social Attitudes (BSA) from 2015 onwards and the Life in Northern Ireland (LNI) survey (LNI) . More details on the BSA weight can be found at [bsa.natcen.ac.uk](https://natcen.ac.uk/british-social-attitudes).

2. *Sampling weight*: this weight adjusts for selection probabilities used in the sampling process and all non-response and/or attrition that occurs after the recruitment surveys but prior to sampling. First, a logistic regression model was created to derive non-response weights to adjust for non-response that occurred prior to sampling, i.e. at the Panel recruitment stage plus any subsequent attrition. The following variables were used as predictors in the model: age and sex groups, region, household type, household income, education level, ethnicity, tenure,

social class group, economic activity, political party identification, and interest in politics. The non-response weight was the inverse of the probability of joining/remaining in the Panel.

As described in the article, a random subsample of panel members was selected for this pharmacogenomics survey. Weights were used to adjust the probabilities of selection, therefore a ‘sample selection’ weight was computed to account for these differential selection probabilities (equal to the inverse of the probability of being selected for the sample), including the over-sampling in Wales and Northern Ireland. The final ’sampling weight’ is the product of the recruitment survey weight, the panel non-response weight and the sample selection weight.

3. *Survey weight*: this weight adjusts for non-response to this specific pharmacogenomic survey.

A logistic regression model was used to estimate the probability of response for each panellist issued to the survey. The Panel survey weight was equal to the inverse of the probabilities of response. This weight adjusts for non-response using the same variables as used for the panel recruitment weight above i.e. age and sex groups, region, household type etc. The resulting survey weight was multiplied by the sampling weight to create the final set of weights. The weights for LNI and BSA respondents were scaled before they were combined so that the proportion of respondents from the four nations of the UK is in line with the UK population.
