# Supplementary material for: A United Kingdom nationally representative survey of public attitudes towards pharmacogenomics
Source: QJM. 2025 Feb 20;118(6):423–33. doi: 10.1093/qjmed/hcaf035 (PMC12419794; doi:10.1093/qjmed/hcaf035)
Supplement: hcaf035_Supplementary_Data [file hcaf035_supplementary_data.zip › NatCenQuestions_ForSupplement.docx]

Questionnaire Specification

Experiences around taking medicines

{ASK ALL}

QMULIntro

The next section will be about DNA testing and medicines.

{ASK ALL}

TakeMed

Have you ever taken a medicine that did not work for you?

By this we mean a medicine which didn’t give you the expected relief or improvement.

Please include prescription and non-prescription medicines that were bought or collected from a pharmacy or

supermarket.

1 Yes

2 No

{ASK ALL}

MedsEff

Have you ever had a side effect from a medicine?

Please include prescription and non-prescription medicines that were bought or collected from a pharmacy or

supermarket.

1 Yes

2 No

{ASK ALL}

PresMed

Are you <b>currently</b> prescribed any medicines on a regular basis?

1 Yes

2 No

{ASK IF PresMed = 1}

PresMedNum

How many different medicines are you <b>currently</b> prescribed?

Range 0…50

SOFTCHECK: IF PresMedNum GT 19: “You have said that you are <b>currently</b> prescribed {PresMedNum}

different medicines. Please check your answer before continuing.”

Knowledge about responses to medicines

{ASK ALL}

MedVar [COLLAPSIBLE GRID; RANDOMISE ROWS; FLIP SCALE]

For each of the following, do you think the statement is true or false?

WEB: “Please select one answer in each section below”

TEL: “INTERVIEWER: READ OUT EACH STATEMENT AND THE ANSWER CODES. REPEAT ANSWER

CODES AS REQUIRED.”

GRID ROWS

1 Everyone responds to medicine in the same way

2 Differences in DNA can be used to predict who is more likely to benefit from a particular medicine

3 Differences in DNA can be used to predict who is more at risk of having a bad reaction to a

particular medicine

GRID COLS

1 True

2 False

3 Don’t know

DNA testing– concept introduction

{ASK ALL}

TestIntro

IF WEB: “Before proceeding, please read the information below about DNA and medicines:”; IF TEL: I will now

read out some information about DNA and medicines…”

[FORMATTING: START BOX, PALE GREY BACKGROUND]

Your body contains DNA which is inherited from your parents and doesn’t change throughout your life.

Your DNA affects how you respond to medicines. Differences in the makeup of your DNA can:

• make you more or less likely to benefit from a medicine;

• affect your risk of getting side effects from a medicine.

Completing a DNA test using a bit of your spit or blood could allow medical professionals to better understand

how you may <b>respond to medicines</b>. The results could be used to help them decide which medicines

and doses would be best for you.

DNA testing to predict medicine response is sometimes referred to as <b>‘personalised prescribing’</b>.

[FORMATTING: END BOX]

[DISPLAY]

{ASK ALL}

QnrFocus

The next set of questions are about <b>DNA testing to predict medicine response</b>.

Note that the results of this DNA test would <b>only</b> provide information about medicine response, and not

disease diagnosis or risk of medical conditions.

DNA testing – Overall interest/willingness in taking test and/or benefits of test

{ASK ALL}

TestMotiv [RANDOMISE 1…5]

Which, if any, of the reasons listed below would make you decide to complete a DNA test to predict medicine

response?

WEB: “Please select all that apply”

INTERVIEWER: READ OUT EACH OPTION AND CODE ALL THAT APPLY

1 Improving how well a medication works for you

2 Reducing the possibility of a bad reaction to a medication

3 Improving the likelihood of you sticking with a medication over the long-term

4 Reducing the number of medications prescribed to you

5 Improving your confidence in a medication chosen for you

6 None of the above [EXCLUSIVE]

{ASK IF MORE THAN ONE OPTION SELECTED AT TestMotiv}

TestMotivMain [RANDOMISE 1…5]

And which of these would motivate you <b>most</b> to complete a DNA test to predict medicine response?

WEB: ""

TEL: “INTERVIEWER: READ OUT”

NOTE TO PROGRAMMER: ONLY DISPLAY ANSWERS SELECTED AT [TestMotiv]

1 Improving how well a medication works for you

2 Reducing the possibility of a bad reaction to a medication

3 Improving the likelihood of you sticking with a medication over the long-term

4 Reducing the number of medications prescribed to you

5 Improving your confidence in a medication chosen for you

{ASK ALL}

PersPres [FLIP SCALE]

Imagine you were prescribed a medicine that had been personalised based on your DNA test results.

Would you be more or less likely to take the medicine as prescribed, compared to if the medicine hadn’t been

personalised?

WEB: ""

TEL: “INTERVIEWER: READ OUT”

1 A lot more likely

2 Somewhat more likely

3 Neither more nor less likely

4 Somewhat less likely

5 A lot less likely

{ASK ALL}

NHSWhoOff [COLLAPSIBLE GRID: RANDOMISE ROWS; FLIP SCALE]

Imagine the NHS were to offer DNA testing to help predict how people would respond to medicines.

How much do you agree or disagree that the NHS should offer this testing to the following groups…?

WEB: “Please select one answer in each section below”

TEL: “INTERVIEWER: READ OUT EACH STATEMENT AND THE ANSWER CODES. REPEAT ANSWER

CODES AS REQUIRED.”

GRID ROWS

1 Those who have several health problems and need to take many medicines

2 Everyone at any age

GRID COLS

1 Strongly agree

2 Agree

3 Neither agree nor disagree

4 Disagree

5 Strongly disagree

{ASK ALL}

PatientInfo [COLLAPSIBLE GRID: RANDOMISE ROWS; FLIP SCALE]

How important do you think it is that patients are made aware of the following pieces of information before they

decide whether to complete a DNA test for medication response?

WEB: “Please select one answer in each section below”

TEL: “INTERVIEWER: READ OUT EACH STATEMENT AND THE ANSWER CODES. REPEAT ANSWER

CODES AS REQUIRED.”

GRID ROWS

1 The aim of this DNA test is to make medicines safer and more effective for them

2 The DNA test to personalise prescribing is a form of genetic testing

3 Even after completing the DNA test they may still have a bad reaction to a medication

4 Even after completing the DNA test a medication may not work for them

GRID COLS

1 Very important

2 Fairly important

3 A little important

4 Not at all important

DNA testing – Resources availability

{ASK ALL}

TestResource

Have you come across any patient materials which explain DNA testing for personalised prescribing?

Materials might include leaflets or websites explaining how it works and why it is done.

1 Yes

2 No

{ASK ALL}

TestResourceFut [MULTICODE: RANDOMISE 1..10]

How would you like information about DNA testing for personalised prescribing to be made available?

WEB: “Please select all that apply”

INTERVIEWER: READ OUT EACH OPTION AND CODE ALL THAT APPLY

1. Leaflets from GP surgery or hospital clinic

2. Posters at GP surgery or hospital clinic

3. Podcasts

4. Online videos

5. On social media such as TikTok, Instagram, Facebook, X (formerly Twitter)

6. Via WhatsApp groups

7. At places of worship

8. At community centres

9. At schools

10. At shopping malls

11. Other (please specify)

12. I do not want this information

DNA testing – Data storage/usage and general concerns

{ASK ALL}

DataStorage [COLLAPSIBLE GRID: RANDOMISE ROWS; FLIP SCALE]

{IF Web “Below are”; IF TEL “I will now read out”} some statements relating to the storage and availability of

results from DNA testing done to predict how people would respond to medicines. The results of such tests do

<b>not</b> provide any other information such as disease diagnosis or risk of conditions.

How much do you agree or disagree with the following statements about results from a DNA test to predict

medicine response?

WEB: “Please select one answer in each section below”

TEL: “INTERVIEWER: READ OUT EACH STATEMENT AND THE ANSWER CODES. REPEAT ANSWER

CODES AS REQUIRED.”

GRID ROWS

1 The results should be stored on my medical record so that it’s available to help prescribe the right

medication for me in the future

2 A copy of the results should be provided to me

3 The results should be available on the NHS app (which can be accessed by phone or computer)

GRID COLS

1 Strongly agree

2 Agree

3 Neither agree nor disagree

4 Disagree

5 Strongly disagree

{ASK ALL}

Worries [FLIP SCALE]

How worried, if at all, would you be about doing a DNA test to personalise your medicine?

WEB: ""

TEL: “INTERVIEWER: READ OUT”

1 Very worried

2 Fairly worried

3 A little worried

4 Not at all worried

{ASK ALL}

Concerns1 [COLLAPSIBLE GRID; RANDOMISE ROWS; FLIP SCALE]

IF WEB: ‘Below are’ IF TEL: ‘I will now read out’ some concerns that people may have about how their medical

data could be used.

Thinking about <b>medical record data such as scan results or blood tests</b>, how worried, if at all, would

you be about the following things?

WEB: “Please select one answer in each section below”

TEL: “INTERVIEWER: READ OUT EACH STATEMENT AND THE ANSWER CODES. REPEAT ANSWER

CODES AS REQUIRED.”

GRID ROWS

1 My privacy being compromised

2 My identity being worked out from the data

3 My ethnicity being identified/being worked out from the data

4 How my family members are related or unrelated to each other being identified/worked out from the

data

5 The data being used for research

6 Police requesting access to the data

GRID COLS

1 Very worried

2 Fairly worried

3 A little worried

4 Not at all worried

{ASK ALL}

Concerns2 [COLLAPSIBLE GRID; RANDOMISE ROWS; FLIP SCALE]

Now thinking about <b>the results of a DNA test for medicine response</b>, how worried, if at all, would you

be about the following things?

WEB: “Please select one answer in each section below”

TEL: “INTERVIEWER: READ OUT EACH STATEMENT AND THE ANSWER CODES. REPEAT ANSWER

CODES AS REQUIRED.”

GRID ROWS

1 My privacy being compromised

2 My identity being worked out from the data

3 My ethnicity being identified/being worked out from the data

4 How my family members are related or unrelated to each other being identified/worked out from the

data

5 The data being used for research

6 Police requesting access to the data

GRID COLS

1 Very worried

2 Fairly worried

3 A little worried

4 Not at all worried

{ASK ALL}

ConcernsOth

Do you have any other concerns, if at all, about completing <b>a DNA test for medicine response</b>, the

results and how they could get used?

OPEN

{ASK ALL}

DataRes [MULTICODE, RANDOMISE 1…5]

If people’s DNA were routinely tested to personalise prescribing, it could potentially be used for research to

make medications safer and more effective and to design new medicines.

Which, if any, of the following groups would you be happy for your DNA test data to be shared with for

<b>research</b> purposes?

WEB: “Please select all that apply”

INTERVIEWER: READ OUT EACH OPTION AND CODE ALL THAT APPLY

1 Health care professionals (e.g., doctors, nurses and pharmacists)

2 The national medicine regulator (the agency responsible for making sure medicines are safe and

work well)

3 Academics or university associated researchers

4 Charities or other non-profit organisations

5 Private companies

6 None of the above [EXCLUSIVE]

MHRA agency

{ASK ALL}

AwareSys

The Medicines and Healthcare products Regulatory Agency (MHRA) is the medicines regulator in the UK. They are responsible for licensing medicines for use in the UK and ensuring that medicines are safe and work well.

Are you aware of the Yellow Card system for reporting side effects from medicines to the UK medicines regulator (the MHRA)?

1 Yes

2 No

{If AwareSys = 1}

AwareSub

Are you aware that any member of the public can submit a Yellow Card report to the regulator to report a bad

reaction that they’ve had to a medication?

1 Yes

2 No

{ASK ALL}

RepInclGD

How much do you agree or disagree with the following statement?

I would like the option to <b>include my DNA test data for medicine response</b> in any report made to the

UK medicines regulator (the MHRA) about a bad reaction I have to a medicine.

WEB: ""

TEL: “INTERVIEWER: READ OUT”

1 Strongly agree

2 Agree

3 Neither agree nor disagree

4 Disagree

5 Strongly disagree
