# Supplementary material for: A United Kingdom nationally representative survey of public attitudes towards pharmacogenomics
Source: QJM. 2025 Feb 20;118(6):423–33. doi: 10.1093/qjmed/hcaf035 (PMC12419794; doi:10.1093/qjmed/hcaf035)
Supplement: hcaf035_Supplementary_Data [file hcaf035_supplementary_data.zip › QJM list of acronyms.docx]

Adverse drug reactions (ADRs)

Pharmacogenomics (PGx)

Cytochrome P450 2C19 (CYP2C19)

Yellow Card (YC)

National Centre for Social Research (NatCen)

British Social Attitudes (BSA)

Life in Northern Ireland (NLI)

The Medicines and Healthcare products Regulatory Agency (MHRA)
